# Microclimate-Controlled Smart Growth Cabinets for High-Throughput Plant Phenotyping

**DOI:** 10.3390/s25247509

**Published:** 2025-12-10

**Authors:** Michael Vernon, Ghazanfar Abbas Khan, Lawrence D. Webb, Abbas Z. Kouzani, Scott D. Adams

**Affiliations:** 1 School of Engineering, Deakin University, Geelong, VIC 3216, Australia; mvernon@deakin.edu.au (M.V.);; 2 Centre for Sustainable Bioproducts, Faculty of Science, Engineering and Built Environment, Deakin University, Geelong, VIC 3216, Australia; 3 School of Life and Environmental Sciences, Deakin University, Geelong, VIC 3216, Australia

**Keywords:** phenotyping, microclimate, growth cabinet, growth chamber, climate change

## Abstract

**Highlights:**

**What are the main findings?**

- A novel, modular, low-cost growth cabinet platform for automated phenotyping is fully described in this work.
- The new platform is validated in both bench-tests and real trials with three different plant species.

**What are the implications of the main findings?**

- This platform enables new climate-change agriculture research, by demonstrating dynamic climate “recipes” to reproduce realistic weather variability.
- The platform also facilitates development of AI-driven crop research via integrated multi-cabinet coordination for synchronised, large-scale experiments.

**Abstract:**

Climate change is driving urgent demand for resilient crop varieties capable of withstanding extreme and changing conditions. Identifying resilient varieties requires systematic plant phenotyping research under controlled conditions, where dynamic environmental impacts can be studied. Current growth cabinets (GC) provide this capability but remain limited by high costs, static environments, and scalability. These limitations pose a challenge for climate change-based phenotyping research which requires large-scale trials under a variety of dynamic climate conditions. Presented is a microclimate-controlled smart growth cabinet (MCSGC) platform, addressing these limitations through four innovations. The first is dynamic microclimate simulation through programmable environmental ‘recipes’ reproducing real climactic variability. The second is interconnected scalable multi-cabinet for parallel experiments. The third is modular hardware able to reconfigure for different plant species, remaining cost-effective at <$10,000 AUD. The fourth is automated data collection and synchronisation of environmental and phenotypic measurements for Artificial Intelligence (AI) applications. Experimental validation confirmed precise climate control, broad crop compatibility, and high-throughput data generation. Environmental control stayed within ±2 °C for 97.42% while dynamically simulating Hobart, Australia, weather. The MCSGC provides an environment suitable for diverse crops (temperature 14.6–31.04 °C, and Photosynthetically Active Radiation (PAR) 0–1241 µmol·m^−2^·s^−1^). Multi-species cultivation validated the adaptability of the MCSGC across *Cannabis sativa* (544.1 mm growth over 34 days), *Beta vulgaris* (123.6 mm growth over 36 days), and *Lactuca sativa* (19-day cultivation). Without manual intervention the system generated 456 images and 164,160 sensor readings, creating datasets optimised for AI and digital twin applications. The MCSGC addresses critical limitations of existing systems, supporting advancements in plant phenotyping, crop improvement, and climate resilience research.

## 1. Introduction

Climate change is accelerating environmental volatility and threatening global food security through increasingly unpredictable growing conditions [1]. Developing crop varieties capable of thriving under volatile conditions requires identifying and selecting adaptive traits through systematic phenotyping. Traditional phenotyping methods, which rely on manual measurement of plant traits (e.g., height, biomass, and leaf colour) are labour intensive and become cost prohibitive when scaled [2,3]. These limitations hinder the development of climate-resilient crop varieties essential for withstanding increased temperature extremes, altered precipitation patterns, and extended drought cycles [3,4]. Global analyses estimate each additional 1 °C of warming reduces wheat yields by approximately 6% [5]. Furthermore, compound drought-heat events can cause losses exceeding 40% [6]. The probability of these co-occurring extremes during growing seasons has increased up to six-fold over recent decades [7]. To overcome these barriers, the following two technological routes are emerging in parallel: Artificial Intelligence (AI) models for automated phenotyping analysis, and automated platforms for high-throughput measurements. Unlike manual methods, such as visual stress identification and disease classification, which can introduce subjectivity and inter-observer variability, automated phenotyping provides more objective, repeatable measurements essential for AI model training [8].

AI is transforming phenotyping by enabling researchers to detect subtle growth pattern changes and analyse large datasets with unprecedented speed and accuracy [4,9,10,11]. Effective model training, however, requires datasets in which phenotypic variation can be attributed to defined conditions. In the field, even plants in close proximity experience different microclimates and exposure histories, breaking this link and preventing the reliable labelling needed for AI-ready datasets. To overcome this, AI models can be trained on datasets generated in controlled environment growth chamber systems. Growth chambers range from compact, reach-in cabinets to walk-in, room-scale facilities; this paper presents a growth cabinet (GC), a modular reach-in system, due to its accessibility, lower cost, and scalability for distributed phenotyping networks. GC systems enable plant growth under repeatable conditions. GC systems enable plant growth under repeatable conditions, producing high-quality phenotyping datasets that are directly comparable and suitable for AI applications [12]. While this solves the repeatability issue, this reproducibility comes at the cost of environmental realism. Most GC systems operate with static temperatures, humidity, and light regimens that fail to reproduce how these systems interact with plant phenotypes in the real-world environment [13]. This disconnect limits their value for climate-resilience research, where identifying adaptive traits requires exposure to more realistic dynamic conditions. Existing GC systems are also constrained by their high costs (€60,000 to €3,000,000 for commercial systems) [14] and isolated operation that restrict high-throughput comparative studies [15,16].

Automated data collection is equally critical, as high-throughput phenotyping requires consistent, high-frequency measurements over entire growth cycles. Current automated phenotyping systems have sought to address this need, but are typically not designed as integrated systems, focusing on measurements generally decoupled from environmental control [17]. These specialist systems generate valuable measurements, but require additional equipment to capture the causal links between the plant performance and the conditions under which it occurs, an integration that is critical for climate-relevant phenotyping [18]. Furthermore, data integration remains a challenge, as temporal synchronisation between phenotyping platforms and environmental systems is essential for capturing developmental dynamics.

An investigation of the current literature was conducted to evaluate how existing solutions address this need for integrated platforms. Papers published after 2014 were identified through targeted searches in Scopus and Google Scholar using combinations of terms including ‘growth cabinet’, ‘growth chamber’, ‘phenotyping’, and ‘controlled environment’. From the literature, studies that specifically described the complete design and implementation of growth cabinet systems were selected, excluding papers that merely reported the use of commercial systems or focused on individual subsystems in isolation. Five representative systems that demonstrated varying approaches to scale, automation, and environmental control were identified. Additionally, one commercial system (Conviron PGW40) was included for comparison with research-developed platforms, though detailed technical specifications for commercial systems proved difficult to obtain from publicly available sources. Table 1 presents a detailed comparison of these six growth cabinet systems.

Physical dimensions of the reviewed GC systems varied significantly, ranging from compact designs restricted to small plant varieties [20], to larger units designed to be installed within environmentally controlled spaces (such as a greenhouse) [19]. Mid-size cabinets were also reported that were targeted specifically towards leafy vegetables [21] while a commercial example was also reviewed which was designed to support taller plants to grow to maturity [24]. The modularity of GC systems was limited. Hatzopoulos et al. [22] included removable sensors and hardware, but most systems, including those by Silva and Machado [19], Ariella et al. [20], and Lee et al. [23] were built around fixed-use cases. While Silva and Machado’s GC [19] was scalable in size, its dependence on greenhouse infrastructure and lack of detail on control architecture limited its standalone utility. Collectively, this illustrates how most GC systems reported in the literature are designed specifically for particular plant species, or experimental contexts, and are not adaptable for different crop needs.

Environmental control capabilities are also heterogeneous across the literature. Temperature and humidity control were all present within the identified studies, but extensions to CO_2_, light quality, and airflow are inconsistent, based on specific use cases [19,20,21,22]. Accuracy is also inconsistently reported and often limited to narrow setpoint windows (e.g., ±1 °C temperature and ±6% relative humidity between 24 and 27 °C [20]). Distributed sensing was only shown in [21,23], which can mask spatial heterogeneity, and limits understanding of cabinet environmental uniformity. Each of the systems evaluated their internal environmental control in different ways, limiting the ability to objectively compare the performance across different systems. The literature shows incremental expansion of controllable environments but an absence of dynamic, closed loop, spatially resolved control with transparent performance reporting across a wide operational envelope.

Dynamic environmental control incorporates diurnal cycles and gradual fluctuations rather than static parameters. Dynamic variation is biologically essential for accurate phenotyping because approximately 30–35% of the plant transcriptome exhibits circadian regulation [25,26]. Plants with circadian rhythms properly synchronised to their environment demonstrate substantial fitness advantages, including more chlorophyll, enhanced carbon fixation, faster growth, and improved survival compared to plants with disrupted rhythms [27]. Moreover, gradual temperature changes activate comprehensive protective mechanisms involving approximately 1600 up-regulated genes that are absent during sudden temperature shifts, with gradual acclimation (22 °C to 45 °C over 6 h) leading to higher survival than step-wise protocols [28]. A 2016 meta-analysis found that phenotyping results obtained from plants grown in the lab correlate rather poorly with those obtained under field conditions (r^2^ = 0.26) [29]. Natural day–night cycles provide both photic and metabolic entrainment signals that coordinate incompatible biochemical processes temporally, optimise resource allocation, and enable anticipatory stress responses, none of which occur under constant or static conditions [25,27].

None of the reviewed GC systems demonstrated dynamic environmental control. They all relied on static setpoints or manual changes, limiting their ability to simulate environments with diurnal or fluctuating stress. Even where feedback loops were applied, using fuzzy or Proportional-Integral-Derivative (PID) control methods, they were used to maintain static setpoints rather than implement dynamic climates [20,21]. This indicates that while feedback control has been explored, dynamic climate simulation remains an unmet need to date.

In parallel with these environmental limitations, automated data collection also shows considerable inconsistency in scope and implementation. Some systems integrated imaging and sensor monitoring [20,22,23], while others relied entirely on manual data collection [19] or only offered partial monitoring without vision systems [21]. A common limitation across all reviewed systems was the absence of native multi-cabinet coordination, hindering high-throughput, multi-variable studies needed for large-scale phenotyping where 10, 20, or more different environments may need to be evaluated to detect and attribute subtle phenotypic differences with confidence.

Cost was another important consideration, with most reviewed systems targeting lower budgets. Most reviewed systems targeted lower budgets with trade-offs in automation or precision [19,20,22,23], while the commercial Conviron PGW40 [24] option, while highly precise, represented a substantially higher investment, beyond the reach of smaller institutions.

Taken together, current systems perform well for single-cabinet, species-specific studies but lack adaptability for large-scale, multi-cabinet, dynamic climate experiments. This highlights a fundamental gap: the absence of a cost-effective, modular platform providing dynamic environmental control while generating synchronised AI-ready datasets. Such datasets also underpin emerging agricultural digital twins, where virtual crop models are continuously updated with real-world measurements to predict growth outcomes [30,31]. However, digital twins require bidirectional data flows linking environmental inputs to plant responses, and Tagarakis et al. [32] found only 6 of 34 surveyed implementations achieved full integration. Controlled environment platforms generating temporally synchronised environmental and phenotypic measurements can address both gaps, enabling AI-driven phenotyping while providing the high-fidelity data streams that predictive models require [31].

The remainder of the paper is structured as follows. Section 2 presents the design and hardware architecture of the proposed MCSGC, followed by describing the experimental methodology used to validate the system performance. Section 3 reports the results obtained from environmental control tests and plant growth experiments. Section 4 discusses implications for digital agriculture and phenotyping research. Section 5 concludes with contributions summary and future research directions.

## 2. Materials and Methods

This section begins by presenting the design and implementation of the MCSGC platform along with the methods used for experimental validation. This is followed by describing the experimental methodology used to evaluate system performance across key GC functions. Baseline performance trial methods are described which evaluate climate control, lighting, water management, dynamic climate testing functionality, while plant growth trials are also described to demonstrate the platforms capabilities in real-world phenotyping trials.

### 2.1. MCSGC Platform

The MCSGC platform incorporates multiple custom instrumented growth cabinets, coordinated by a centralised client–server architecture. Each cabinet has three sensing subsystems; first, the sensing array external to the cabinet that senses the connected water supply parameters including temperature, level, acidity, and conductivity. Second—the internal environmental sensing array senses air temperature, Relative Humidity (RH), CO_2_ concentration, and barometric pressure. Finally, there is a vision-based data collection subsystem for the collection of automated phenotyping measurement that includes high-definition, thermal, and Time-of-Flight (ToF) cameras.

**Figure 1:**
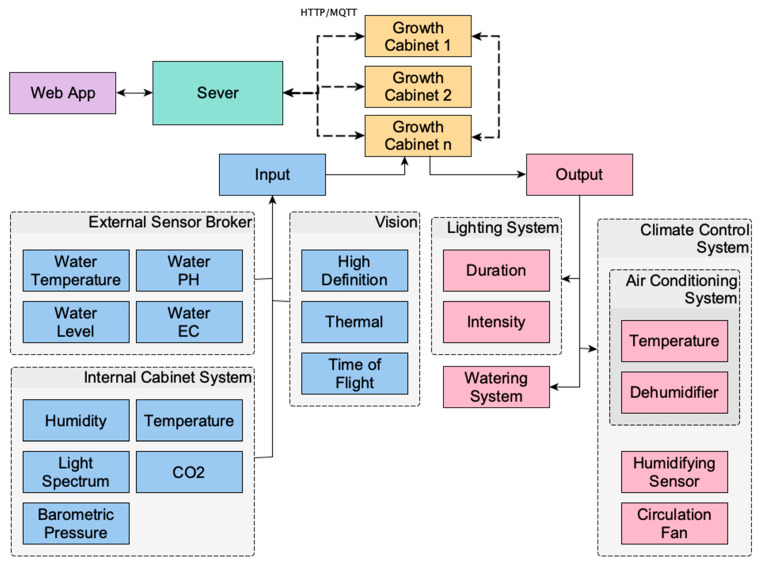
The architecture of the microclimate-controlled smart growth cabinet (MCSGC). Showing system inputs, control subsystems, and interconnections between cabinets, sensors, and the server.

The control subsystems of each cabinet can control air-temperature, dehumidification/humidification, lighting (for both intensity and photoperiod) as well as circulation fans for airflow. All subsystems are managed through the central server allowing each cabinet to function as part of a coordinated, multi-cabinet network. This architecture is shown diagrammatically in Figure 1.

The platform supports a variety of crop types through modular design principles that facilitate flexible sensor integration and experimental customisation. This adaptability overcomes the species-specific constraints of previous systems, enabling the same cabinet infrastructure to be reconfigured for diverse plant phenotypes and research objectives. By streamlining coordinated data capture and maintaining consistent management across multiple cabinets the platform reduces labour demands while producing synchronised, high-quality datasets suitable for AI-driven phenotyping in dynamic climates studies. The following section describes the physical design of the growth cabinets, detailing how the architecture is implemented in practice.

### 2.2. Microclimate-Controlled Smart Growth Cabinet

The cabinet portion of the platform provides the physical foundation and consists of a physical cabinet where the plants, sensors, and vision system are housed, and an integrated array of environmental control and monitoring subsystems.

#### 2.2.1. Physical Design

**Figure 2:**
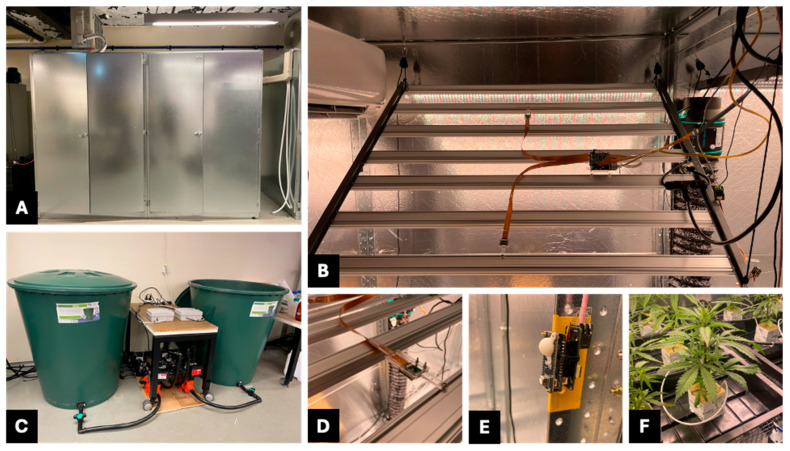
(**A**) Double-sized cabinet with closed doors. (**B**) Top of light bar showing cabinet unit and both camera placements on left hand side of unit. (**C**) Water tank pumps and solenoid system for multi cabinet irrigation. (**D**) Close-up view of camera mounting and placement on light bar. (**E**) Close-up view of internal environmental sensor Printed Circuit Board (PCB). (**F**) Example of plant in growth trays with individual dripper lines.

Each of the MCSGC systems is built from galvanised steel panels with integrated insulation, with a modular frame designed for durability and flexibility; images of this system are shown in Figure 2.

Each of the MCSGC systems is built from galvanised steel panels with integrated insulation, with a modular frame designed for durability and flexibility. The cabinets were selected as commercially available off-the-shelf solutions in multiple sizes, prioritising cost effectiveness and rapid deployment. Foilboard Green 25 mm insulation panels with an R-value of 0.64 K·m^2^/W (Foilboard Australia, Pakenham, Australia) [33], were fixed to the cabinet internal skin to improve thermal performance while maintaining the system cost target of <AUD $10,000. Images of this system are shown in Figure 2. The system in Figure 2A has a footprint of 2100 mm (H) × 2800 mm (W) × 1900 mm (D). Modularity is achieved through removable panels that allow units to be connected or detached along the *X*, *Y,* and *Z* axis. The demonstrated system comprises two sections each measuring 2100 mm (H) × 1400 mm (W) × 1900 mm (D), joined side-by-side; additional sections can be added to expand cabinet size based on experimental requirements or for different crop species. Inside the GC are retractable racks supporting three sub-irrigation trays (Figure 2F), enabling isolated water and nutrient delivery for experimental replicates (Figure 2C). Integrated cable management, water inlets/outlets, and lighting mounting points for flexibility are shown in Figure 2B,D,E.

#### 2.2.2. Environmental and Control Systems

Each MCSGC system features a Raspberry Pi 5 Single-Board Computer (SBC; Raspberry Pi Ltd, Cambridge, UK) with communications and power provided by a Power over Ethernet (POE) through a TL-SG105PE switch (TP-Link Technologies, Shenzhen, China). The controller receives environmental setpoints from the server, collects sensor data, and transmits commands to environmental control subsystems. A separate Raspberry Pi 5 SBC is embedded next to the water supply tank, operating as an independent node to collect data from the water supply.

Each cabinet includes three major subsystems: environmental monitoring, environmental control, and vision-based data collection. These integrated systems maintain environmental parameters required for precise phenotyping.

##### Environmental Monitoring

Input data are captured into the Raspberry Pi’s using Inter-Integrated Circuit (I^2^C), Serial Peripheral Interface (SPI), and Message Queuing Telemetry Transport (MQTT) protocols depending on the sensor. Data are stored locally then transmitted to the growth server via MQTT and Hyper-Text Transfer Protocol (HTTP). Environmental monitoring is achieved by both internal sensors that monitor cabinet conditions and external sensors which monitor the external water supply. Sensors were selected balancing measurement accuracy against cost constraints, prioritising commercially available components with standardised I^2^C/SPI communication protocols to facilitate system replication.

The platform incorporates sensors across three functional categories: internal cabinet environment monitoring (temperature and humidity (Sensirion AG, Stäfa, Switzerland) [34], atmospheric pressure (STMicroelectronics, Geneva, Switzerland) [34], light spectrum (ams-OSRAM AG, Premstätten, Austria) [35], and CO_2_ concentration (Atlas Scientific, Long Island City, NY, USA) [36]), external water supply monitoring (pH; Atlas Scientific, Long Island City, NY, USA) [37], electrical conductivity (EC; Atlas Scientific, Long Island City, NY, USA) [38] and water temperature (Atlas Scientific, Long Island City, NY, USA) [39]), and vision-based phenotyping (detailed in Section 2.2.3). Table S1 in the Supplementary Material provides complete specifications for all environmental sensors, including measurement accuracy, communication interfaces, and subsystem assignments. Together these devices generate a consistent, high-resolution data stream that is used to both control the cabinet conditions in real-time and enable robust post-experiment analysis.

Environmental sensors were factory calibrated by manufacturers with documented accuracy specifications listed in Table S1 in the Supplementary Material. The AS7341 multispectral sensor (ams-OSRAM AG, Premstätten, Austria) used for continuous PAR monitoring was cross-calibrated against a calibrated Apogee Instruments MQ-610 (Apogee Instruments, Logan, UT, USA) quantum sensor positioned at the fixed mounting location, with a correction offset applied to match the reference instrument. Atlas pH and EC probes monitoring the nutrient solution were calibrated following manufacturer guidelines using standard buffer and conductivity solutions.

##### Environmental Control

The environmental control for the GC is designed around three areas including lighting, water, and a climate control system.

Control of the lighting system is achieved through a GP8403 PWM to 0–10 V Signal Converter (DFRobot, Shanghai, China) [40] on a custom Printed Circuit Board (PCB) interfacing via I^2^C connection. This enables MQTT-based remote adjustments of intensity, spectrum, and photoperiod, allowing replication of diurnal cycles or tailored experimental treatments.

Control over the lights is achieved through a GP8403 PWM to 0–10 V Signal Converter [41], which interfaces with a microcontroller via an I^2^C connection. This configuration allows for seamless integration with MQTT protocols, enabling remote and programmable adjustments of the light intensity, spectrum, and photoperiod. The fine-tuned control provided by this system ensures the lighting conditions can replicate diurnal cycles or be tailored to specific experimental treatments, enhancing experimental flexibility. To address thermal management and to ensure a stable internal cabinet temperature, the power supplies are mounted externally to minimise heat buildup within the cabinet.

For water and nutrient delivery, the cabinet employs Netafim PCJ LCNL Drippers (Netafim, Tel Aviv, Israel), with integrated pressure compensation, maintaining a fixed flow rate of 1.2 litres per hour [42]. An Orbit B-Hyve 12-Station Indoor/Outdoor Smart Irrigation Controller (Orbit Irrigation, North Salt Lake, UT, USA) [43] manages watering schedules, frequencies, and durations through a Wi-Fi-enabled interface. Water is supplied by an Orange Pumps HT100-PM Pump (Orange Pumps, Mount Waverley, Australia), which ensures a consistent pressure of 2.5 bar. The nutrient solution is prepared manually and stored in a 500 L tank housed within the climate-controlled facility (ambient temperature ~21 °C), where it is continuously aerated using an air stone to maintain homogeneity.

Climate control uses a Mitsubishi Heavy Industries SRK20ZSXA-W 2 kW Wi-Fi Split System Air Conditioner (Mitsubishi Heavy Industries, Tokyo, Japan) [44], maintaining temperatures between 14 °C and 38 °C (±1.5 °C tolerance). Humidity control employs a Mars Hydro Ultrasonic Cool Mist 15 L Large Humidifier (Mars Hydro, Shenzhen, China) supporting 30% to 80% relative humidity (±5% tolerance). System efficiency is enhanced by reclaiming condensed water from the air conditioner to supply the humidifier. Uniform air distribution is achieved through the split system’s cooling, heating, fan, and dry modes, supplemented by an integrated circulation fan (Mars Hydro, Shenzhen, China).

##### Vision-Based Monitoring

The vision subsystem forms the third critical component of the platform, providing non-invasive monitoring of plant growth and morphology. The system consists of 3 cameras: a 64MP high-definition RGB camera (Arducam, Nanjing, China) [45], a FLIR Lepton 3.5 thermal camera (Teledyne FLIR, Wilsonville, OR, USA) [46], and ToF depth camera (Arducam, Nanjing, China) [47], connected to the cabinet Raspberry Pi to capture complementary datasets for both real-time and post-experiment phenotyping analysis. Complete specifications for all vision-based sensors are provided in Table S2 of the Supplementary Material.

Modularity is a core principle of the MCSGC platform, allowing the cabinets to be tailored to different experimental needs without requiring a system redesign. This flexibility is supported by the modular sensor framework built on top of interchangeable sensors, rather than fixed, custom-designed subsystems, which contrasts with existing systems that require hardware-specific drivers and have fixed sensor configurations. As a result, the platform can expand monitoring capabilities with minimal effort, ensuring that platform remains versatile for researchers’ diverse needs.

#### 2.2.3. Software

**Figure 3:**
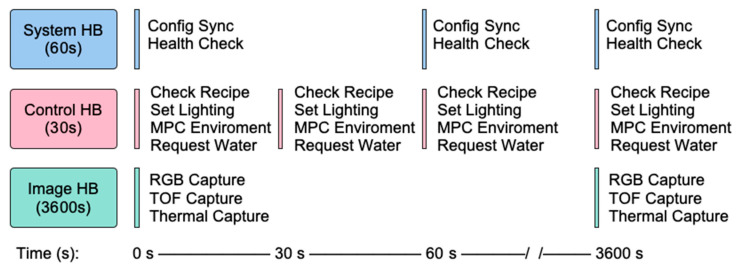
System software flow diagram showing three asynchronous heartbeat sequences operating at 30 s (control), 60 s (system), and 3600 s (image) intervals during continuous operation.

The MCSGC software architecture is designed to give simple yet fine-grained control over the environmental conditions and data collection across multiple cabinets. At the start of an experiment, the researcher creates an environmental “recipe” as a Comma Separated Value (CSV) file and uploads it to the central server. This allows recipes to be easily edited, reviewed, and shared. This CSV is sorted into a schedule of setpoints, where users can set the lighting, temperature/humidity, and watering at intervals as fine as 30 s. The server validates and stores this recipe, and the user can select which cabinets to apply it to and when to start the experiment. Once the experiment is started, each cabinet implements three “heartbeat” processes. The system heartbeat (default every 60 s) maintains connectivity with the server and performs connectivity health checks. The control heartbeat (default every 30 s) checks the CSV “recipe” against the current environment of the cabinet and changes if required. The image heartbeat (default 60 min) captures the plant phenotyping images and uploads them to the central server. Together, these asynchronous processes provide continuous control, reliable data capture, and seamless coordination across multiple cabinets. The aggregated data, including images, environmental readings, and recipe settings are stored on the server for later analysis. Details of the growth server operation is provided in the Supplementary Material. The full system heartbeat flow can be seen in Figure 3.

Temperature regulation within the cabinet employs a Model Predictive Control (MPC) algorithm [48] that continuously learns thermal response characteristics through monitoring temperature changes under different operating modes: cooling, heating, fan-only operation, and idle conditions. The control algorithm updates thermal response parameters using an exponential moving average:

(1) rate_new=(1−α)×rate_old+α×observed_rate,

where α = 0.1 represents the learning rate.

For each 10 s control cycle, the system:

1. Measures current temperature and humidity
2. Predicts future temperature:

(2) T(t+Δt)=T(t)+rate_k×Δt,

where T(t) is the current temperature, rate_k is the learned thermal response rate for the current operating mode k, and Δt is the prediction time step

3. Selects optimal control action based on predicted deviation from set
4. Implements early termination when approaching target temperature to prevent overshoot

The optimisation minimises the cost function:

(3) $$J=\Sigma (w_{1}(T(t+i)-T\_sp{)}^{2}+w_{2}(\Delta P{)}^{2}),$$

where J is the cost function, w_1_ and w_2_ are weighting coefficients, T(t + i) is the predicted temperature at time t + i, T_sp is the target setpoint, and ΔP is the change in control action.

Dynamic control parameters include prediction horizons (30–90 s), minimum operating times, and stabilisation periods between heating and cooling modes to protect equipment and improve efficiency.

All cabinet software was developed in Python 3.11.2 (Python Software Foundation, Wilmington, DE, USA) running on Raspberry Pi OS based on Debian 12 ’Bookworm (Raspberry Pi Ltd, Cambridge, UK). The MPC algorithm employs NumPy v2.3.2 for numerical computation. The central server architecture uses FastAPI v0.116.1 as the RESTful API framework, with all experimental data, environmental readings, and imaging datasets stored in a PostgreSQL v15.13 relational database (PostgreSQL Global Development Group).

### 2.3. Climate Control

The climate control subsystem was evaluated to characterise operational temperature boundaries, static setpoint stability, and validate dynamic environmental control required for multi-species plant research.

#### 2.3.1. Temperature Range

Operational temperature boundaries of the MCSGC were determined through systematic ramp testing to establish the practical operating envelope for experimental design. Two one-hour ramp tests challenged the system limits using extreme set-points: a minimum test targeting 1.0 °C (below the AC controller’s 16 °C design limit) to determine the true lower boundary and a maximum test targeting 50.0 °C (above the AC controller’s 32 °C design limit) to establish the upper operational limit.

These tests quantified cooling and heating rates under controlled conditions, characterised time to stabilisation, and measured steady-state temperature fluctuation. Success criteria required determination of achievable temperature boundaries with associated thermal response rates, providing parameters for system specification and experimental planning.

#### 2.3.2. Static Temperature Setpoint

Stable set-point control is essential for experiments requiring consistent environmental conditions, such as long-term growth trials or studies isolating specific variables. Verifying the system’s ability to maintain a fixed temperature ensures minimal fluctuation and high repeatability between experiments. Initial testing characterised the system’s ability to maintain a static temperature set-point over a one-hour period. Temperatures of 23 °C and 38 °C were selected to show the performance at a medium and high temperature. This test evaluated the stability of the control system and verified the precision of temperature maintenance under steady-state conditions, with performance assessed through deviations from the programmed set-point and variability over the test period.

#### 2.3.3. Dynamic Recipes—Weather Simulation

Dynamic environmental control capabilities were validated through replicating a real-world weather simulation to demonstrate the system’s recipe functionality.

Weather data were sourced from the Australian Bureau of Meteorology (BOM) station in Hobart for 8–11 March 2025 [49]; these data had diurnal temperature fluctuations between 15.3 °C and 30.3 °C with 30 min fidelity. The 48 h simulation protocol evaluated the system’s ability to track programmed temperature transitions and maintain accuracy during dynamic set-point changes, with performance evaluated through tracking accuracy, deviation from setpoints, and responsiveness.

### 2.4. Lighting Management

This experiment aims to validate the LED array system’s ability to deliver precise, repeatable Photosynthetically Active Radiation (PAR) levels. Spatial homogeneity was first assessed using a calibrated Apogee Instruments MQ-610 quantum sensor positioned at 5 measurement points across the growing area at 1.20 m height. Measurements showed PAR uniformity within ±9.22% across the cabinet (1162–1280 μmol·m^−2^·s^−1^ range). To manage spatial variation, cabinet interiors incorporated reflective foil board panels that improved light distribution. The AS7341 multispectral sensor was subsequently calibrated against the Apogee reference metre at its fixed mounting position for continuous monitoring.

For characterisation testing, cabinet doors remained closed throughout to eliminate ambient light interference. Baseline measurements confirmed zero ambient PAR contribution before testing commenced. The characterisation protocol involved systematic PWM signal incrementation in 1% steps from 0 to 100% full-scale voltage, with PAR measurements recorded at each increment. Each measurement point allowed 5 s stabilisation after PWM adjustment to ensure steady-state conditions, eliminating transient responses from the LED drivers. Performance was evaluated by analysing linearity of PAR output with regard to input, as well as maximum and minimum achievable levels.

### 2.5. Water Management

This experiment validates the irrigation system’s temporal control accuracy and volumetric consistency, confirming its ability to deliver controlled volumes from minimal amounts for water-limited experiments (15 s pulses) to sustained irrigation for high-transpiration conditions (300 s durations). The characterisation aimed to establish reliable flow rate profiles across seven duration intervals (15, 30, 60, 120, 180, 240, and 300 s).

The testing sequence involved pre-test system priming with 5 s activation to ensure uniform starting conditions, followed by collection vessel positioning directly beneath dripper outlets. Programmatic activation for each specified duration was followed by immediate volume measurement and flow rate calculation. Measurement precision employed graduated cylinders with ±0.1 mL precision for volumes ≤25 mL (15–60 s tests) and 100 mL cylinders with ±1 mL precision for larger volumes, with 5 mL syringe verification for overflow measurement. Each dripper underwent individual testing across all seven duration intervals, with three replicates per duration to assess consistency. Performance was evaluated by determining linearity and flow-rate consistency.

### 2.6. Plant Cultivation

Plant growth trials were conducted to demonstrate and evaluate the platform’s practical application as a stable plant-growth environment for phenotyping studies. Initial validation experiments were performed across three species (one trial per species) with contrasting morphological and environmental requirements to comprehensively test system capabilities: *Cannabis sativa*, *Beta vulgaris* subsp. *vulgaris* (silverbeet), and *Lactuca sativa* (lettuce).

Environmental parameters described in Table 2 were selected based on reported optimal ranges for each species: *C. sativa* vegetative growth at 24–30 °C, 18–22 °C during flowering, optimal RH range of 50–60% [50] and 600–900 μmol·m^−2^·s^−1^ [51], *B. vulgaris* subsp. vulgaris as a cool-season leafy crop at 14–21 °C [52] and 200–250 μmol·m^−2^·s^−1^ [53], and *L. sativa* optimal conditions at 22–25 °C and 200–250 μmol·m^−2^·s^−1^ [54]. This selection challenged the platform across temperature (18–24 °C), light intensity (200–600 μmol·m^−2^·s^−1^), humidity (50–65% RH), and irrigation frequency (manual to 4× daily).

Growth configurations varied to demonstrate platform modularity. *C. sativa* employed dual cabinets with six trays containing seven plants per tray (forty-two plants total), testing multi-cabinet coordination. *L. sativa* used a single cabinet with three plants per tray (nine plants total), validating minimum viable experiment size. *B. vulgaris* utilised three trays with 20 plants per tray (60 plants total), providing manual irrigation comparison. Growth media varied between rockwool (*C. sativa*) for precise nutrient control and soil (*B. vulgaris*, *L. sativa*) representing typical horticultural practice. All *C. sativa* specimens were cultivated under Victorian Authority for Low THC Cannabis (Authority number 2024/1, Agriculture Victoria).

The platform’s growth monitoring capability was validated through imaging analysis and environmental data collection. Continuous operation of the cabinet ensured stable image capture and sensor data logging, with environmental parameters and growth media successfully customised for each crop. Plant height was quantified with the cabinet ToF camera paired with the high-definition camera that captured amplitude and RGB images at set intervals. Height (mm) was calculated using:

(4) $$Height\left(mm\right)=maxRange\times \left(1-\frac{pixelValue}{255}\right),$$

where maxRange is the camera’s maximum sensing distance (set at 2000 mm) and pixelValue is the mean intensity of the selected region.

Total growth was determined by comparing the plant region at the start and end of the growth experiment using the ToF images without disturbing the plants. RGB imagery provided visual confirmation of these volumetric growth trends.

Performance was evaluated by comparing observable growth across all species, quantifiable growth measurements, stable maintenance of environmental parameters, and comprehensive data collection throughout growth cycles. Statistical analysis employed growth rate calculations, environmental parameter standard deviations, data collection completeness metrics, and Leaf Area Index (LAI) growth comparisons over time.

### 2.7. Environmental Data Collection and Management

Data collection performance was evaluated during *Lactuca sativa* cultivation over a 19-day growth period to validate the system’s automated monitoring capabilities. Sensor readings for temperature, relative humidity, and PAR intensity were recorded at 10 s intervals and stored locally in CSV format via the cabinet’s IoT controller. High-definition images were captured at fixed hourly intervals using the integrated camera system, while TOF (Time-of-Flight) depth measurements were taken concurrently to enable 3D growth estimation.

All recorded data streams were time-stamped using the system’s synchronised clock, allowing alignment of environmental parameters with phenotypic measurements. The control server automatically appended metadata, including cabinet ID, recipe version, and experiment identifier, to each dataset. Imaging datasets were stored in lossless PNG format to preserve detail for phenotyping analysis. TOF raw depth maps and point-cloud data were retained in binary format, while derived height estimates were computed using the growth monitoring algorithm described in Section 2.4. The standardised CSV and PNG formats facilitate automated analysis through Python-based workflows and are compatible with machine learning frameworks for AI-driven phenotyping applications.

### 2.8. Power Consumption

The system’s power consumption was characterised across lighting and climate control subsystems to quantify operational energy requirements. Understanding power draw enables researchers to estimate operational costs and optimise energy efficiency for extended phenotyping trials.

Power consumption was measured at the mains supply, with voltage recorded for each measurement. System baseline power (controllers, sensors, communications) was first established with lighting at 0% intensity and climate control disabled. Lighting power was then characterised by incrementally increasing intensity through minimum activation threshold, 25%, 50%, 75%, and 100% levels. Climate control, provided by the split system air conditioner, was subsequently isolated and measured independently across three operating modes: cooling, heating, and fan-only circulation. Each measurement was recorded after 60 s stabilisation to ensure steady-state conditions. Thermal efficiency was calculated as the ratio of temperature change rate (°C·min^−1^) to power consumption (kW), normalised to the 11.17 m^3^ cabinet volume. Performance was evaluated by analysing power scaling linearity with lighting intensity and comparative efficiency across climate control operating modes.

## 3. Results

The results are presented to demonstrate both the baseline performance of the MCSGC platform and its application in plant cultivation trials. The following subsections present the outcomes of the experiments described in Section 2.

### 3.1. Climate Control

Temperature control was evaluated through operational range tests, static setpoint maintenance, and dynamic climate tracking protocols described in Section 2.3.

#### 3.1.1. Temperature Range

**Figure 4:**
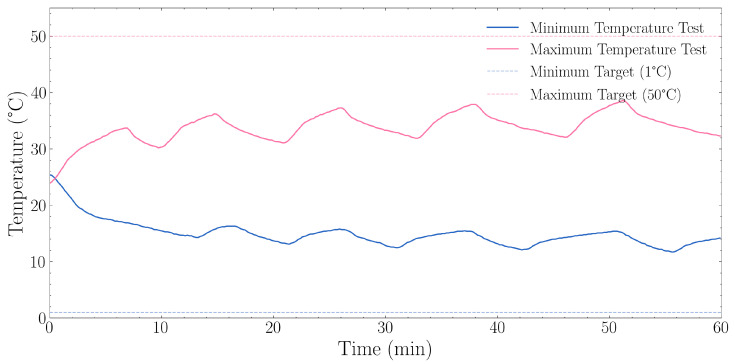
Temperature range performance during minimum (1 °C target) and maximum (50 °C target) temperature tests over a 60 min period. The blue solid line represents the minimum temperature test with its corresponding dashed line showing the 1 °C target. The orange solid line represents the maximum temperature test with its corresponding dashed line showing the 50 °C target. The minimum temperature test achieved a final temperature of 13.7 °C (last 10 min average) with a lowest point of 11.7 °C, resulting in an offset of +12.7 °C from the target. The maximum temperature test achieved a final temperature of 35.2 °C (last 10 min average) with a highest point of 38.6 °C, resulting in an offset of −14.8 °C from the target.

The results of the minimum and maximum temperature experiment as described in 2.3.1 can be observed in Figure 4. Starting from 25.4 °C ambient temperature, the system achieved a rapid decrease to 11.7 °C with an average cooling rate of 0.246 °C·min^−1^ during this initial phase. The system’s cooling performance demonstrated a non-linear cooling curve, with faster initial cooling rates that gradually diminished as temperature approached the system’s physical limit. During the final 10 min of the one-hour period, the mean temperature stabilised around 13.7 °C. The lower temperature bound was determined to be 11.7 °C.

The maximum temperature test results are described in Section 2.3.1, starting from 38.6 °C with an average heating rate of 0.287 °C·min^−1^. The test revealed that the heating system provides relatively linear temperature increases with consistent performance throughout the range. During the final 10 min of the one-hour test, the mean temperature stabilised at approximately 35.2 °C. The system achieved and maintained temperatures in the 35–40 °C range, while notably below the 50 °C setpoint, which was intentionally set outside the air conditioner’s range.

#### 3.1.2. Static Temperature Setpoint

**Figure 5:**
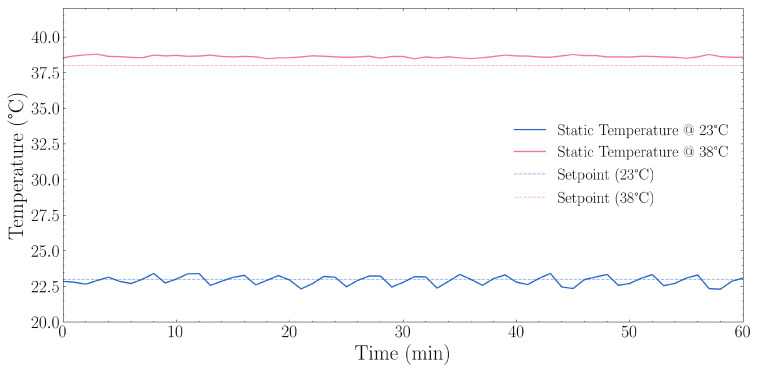
Static temperature stability performance at two setpoints (23 °C and 38 °C) over a 60 min period. The blue solid line shows temperature measurements at the 23 °C setpoint with its corresponding dashed line indicating the target. The orange solid line shows temperature measurements at the 38 °C setpoint with its corresponding dashed line indicating the target. At the 23 °C setpoint, the system maintained a mean temperature of 22.92 °C ± 0.319 °C standard deviation (SD) with a range of 22.31–23.42 °C, achieving an offset of −0.08 °C from the target. At the 38 °C setpoint, the system maintained a mean temperature of 38.62 °C ± 0.072 °C SD, with a range of 38.47–38.79 °C, achieving an offset of +0.62 °C from the target.

The 23 °C static setpoint test results, described in Section 2.3.2, are shown in Figure 5. The system maintained a mean temperature of 22.92 °C with a standard deviation of 0.319 °C. The total temperature range observed was 1.11 °C (between 22.31 °C and 23.42 °C), with the system maintaining temperature within ±0.56 °C of the mean.

With a static setpoint of 38 °C as described in Section 2.3.2, the results are also as observed in Figure 5. The system demonstrated higher stability with a standard deviation of only 0.072 °C, approximately 4.4 times lower than at ambient temperature. The total temperature range was just 0.32 °C (between 38.47 °C and 38.79 °C).

#### 3.1.3. Dynamic Recipes—Weather Simulation

**Figure 6:**
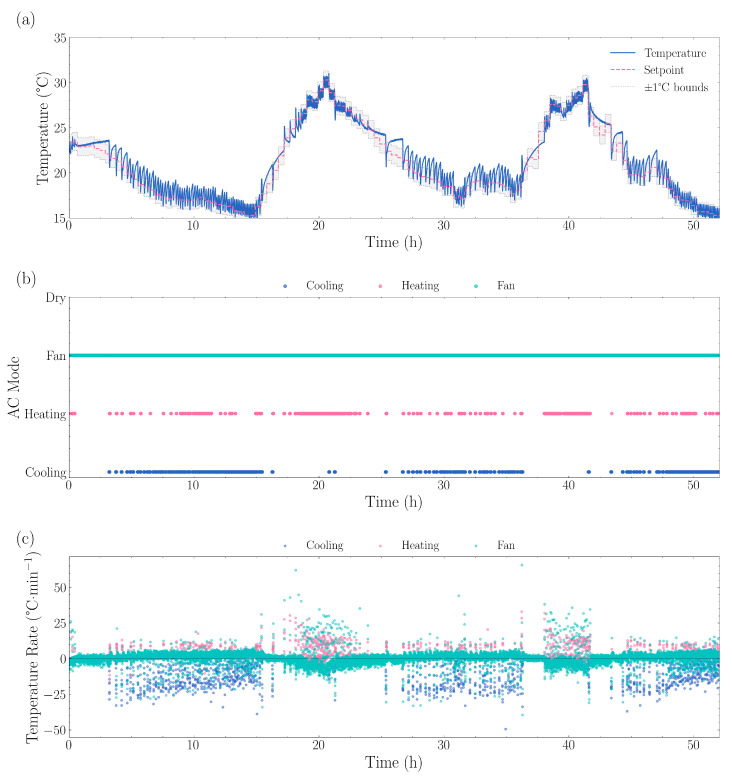
Dynamic temperature control performance showing (**a**) measured temperature tracking versus setpoint, with the grey shaded region indicating ±1 °C bounds, (**b**) air conditioner operating mode transitions between cooling, heating, fan, and dry modes, and (**c**) temperature change rates by mode. The system demonstrated cooling rates of −9.97 °C·min^−1^, heating rates of +6.80 °C·min^−1^, and fan-only rates of +0.57 °C·min^−1^. Mode usage consisted of 83.6% fan mode, 9.6% cooling mode, and 5.7% heating mode, with 678 total mode transitions averaging 0.4 min between changes. The average absolute deviation from setpoint was 0.74 °C, with positive deviations (too warm) averaging +0.90 °C and negative deviations (too cool) averaging −0.44 °C.

The 48 h Hobart weather simulation results are presented in Figure 6. A temperature range of 14.60 °C to 31.04 °C was observed. Throughout the test, the system maintained a mean absolute error of 0.76 °C between the actual temperature and setpoint. The maximum positive deviation recorded was 3.34 °C, with a maximum negative deviation of −2.41 °C, occurring during transition phases as the system adjusted to new setpoints.

During stable periods when the setpoint remained constant, the standard deviation of temperature error was 0.84 °C. Temperature control performance analysis showed the system maintained temperatures within ±0.5 °C of setpoint 42.5% of the time, within ±1.0 °C for 67.1% of the time, within ±1.5 °C for 86.7% of the time, with large deviations exceeding ±2.0 °C occurring in only 2.58% of all measurements.

The system typically required 6.69 min on average to reach within 0.5 °C of a new setpoint, with response times ranging from 0.21 to 61.11 min depending on the magnitude of the transition. The system exhibited rapid response characteristics, achieving cooling rates of 0.78 °C·min^−1^ and heating rates of 0.52 °C·min^−1^, with mode transitions occurring every 4.9 min on average. Thermal analysis indicated estimated times of 2.6 min to cool by 2 °C and 3.9 min to heat by the same amount.

### 3.2. Lighting Management

**Figure 7:**
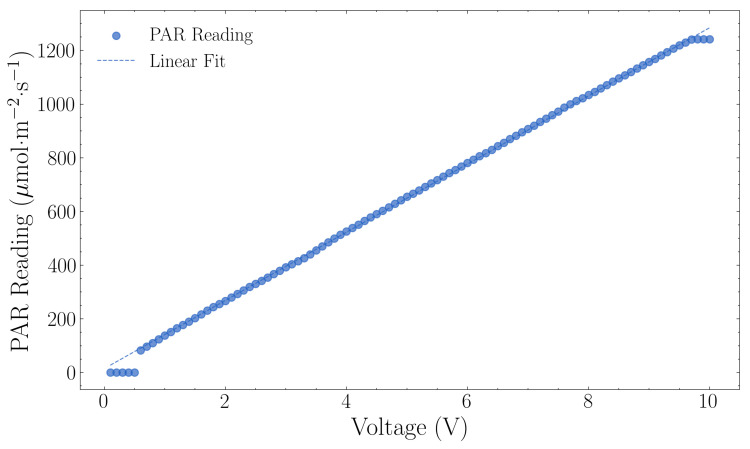
Lighting sensor calibration showing photosynthetically active radiation (PAR) measurements versus voltage (0–10 V pulse width modulation). The linear regression yielded a calibration factor of 127.00 μmol m^−2^ s^−1^ per volt with R^2^ = 0.9996.

Figure 7 shows the PAR output characterisation results as outlined in Section 2.2. The LED array activated when the PWM signal exceeded 0.6 V, providing an initial output of 83 μmol·m^−2^·s^−1^ (Figure 7). PAR output increased linearly with voltage, with each 1 V increase corresponding to 127.65 μmol·m^−2^·s^−1^. The system reached saturation at 9.7 V, producing a maximum output of 1241 μmol·m^−2^·s^−1^.

Within the active control range, from 0.6 V to 9.7 V, the linear relationship can be described by the following equation:

(5) $$PAR=127.65\times Voltage+12.15,\;R^{2}=0.9999,$$

The coefficient of determination (R^2^ = 0.9999) confirmed linear control throughout the active range of the lighting system.

### 3.3. Water Management

**Figure 8:**
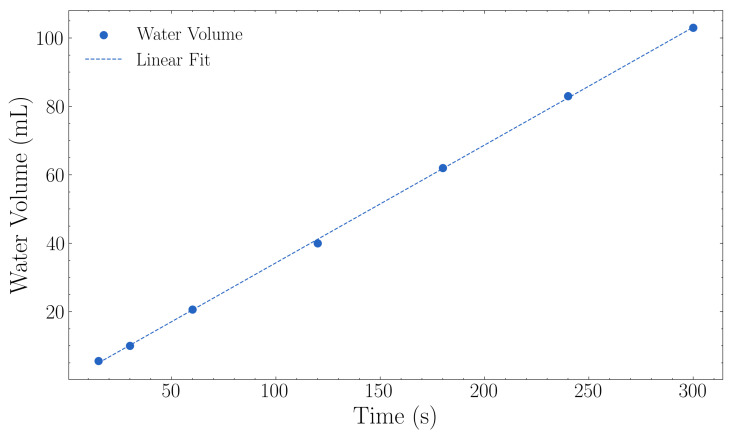
Water flow calibration showing measured water volume versus time. The linear regression yielded a flow rate of 0.34 mL·s^−1^ (1.24 L·h^−1^) with R^2^ = 0.9998.

Figure 8 presents the irrigation flow rate characterisation results as described in Section 2.3. The Netafim PCJ drippers delivered an average flow rate of 1.24 L·h^−1^ with a variation of ±0.09 L·h^−1^ (approximately 0.025 mL·s^−1^) across all tested durations (Figure 8). The measured flow rate closely matched the manufacturer’s specification of 1.2 L·h^−1^.

The linear relationship between the control signal (u), representing the PWM duty cycle applied to the valve, and the resulting flow rate was:

(6) $$Flow\;Rate\left(L/hr\right)=0.34\times u-0.16,\;R^{2}=0.9998$$

The coefficient of determination (R^2^ = 0.9998) demonstrated linear control between the control signal and measured flow rate across the operational range.

### 3.4. Plant Cultivation

**Figure 9:**
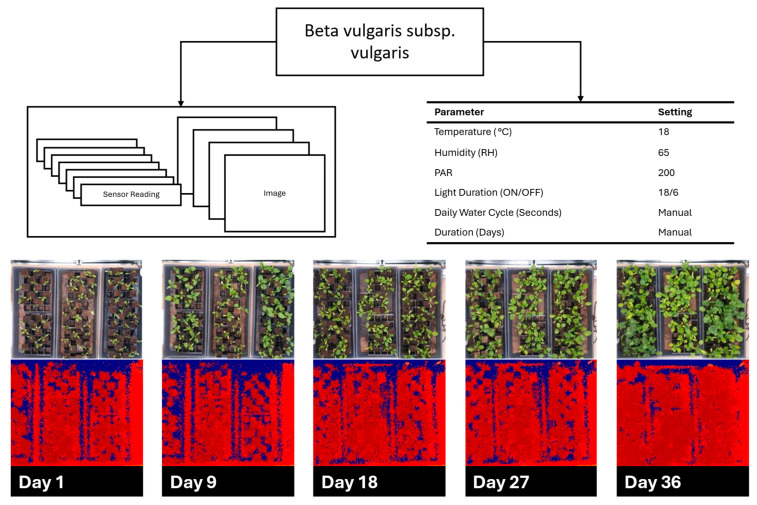
*Beta vulgaris* subsp. vulgaris cultivation over 36 days demonstrating environmental parameters with red, green, blue (RGB), and time-of-flight (TOF) images spread out over the growth cycle.

**Figure 10:**
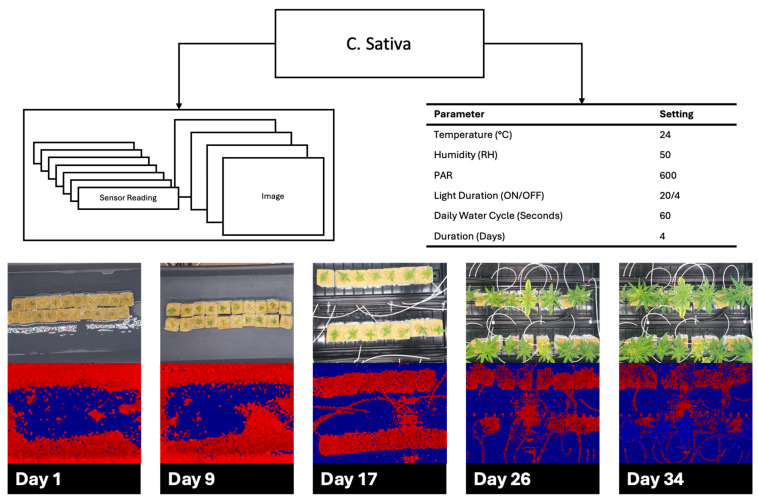
*Cannabis sativa* cultivation over 34 days demonstrating environmental parameters with red, green, blue (RGB), and time-of-flight (TOF) images spread out over the growth cycle.

Figure 9 and Figure 10 show the cultivation results for *B. vulgaris* and *C. sativa* respectively, as described in Section 2.4. *B. vulgaris* exhibited a height increase of 123.6 mm over 36 days. *C. sativa* specimens showed an increase of 544.1 mm over 34 days. Visual inspection of RGB images confirmed these measurements and documented leaf development and plant architecture changes throughout the growth periods.

**Figure 11:**
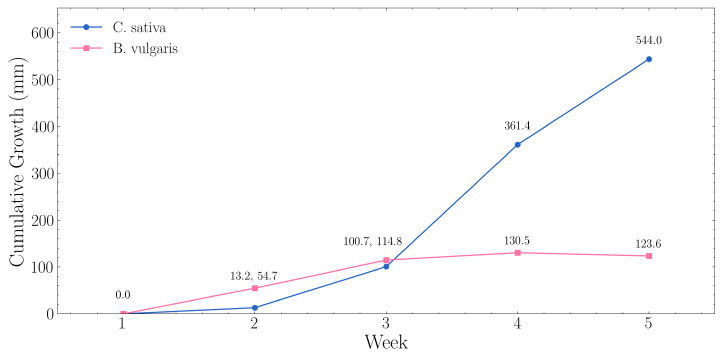
Weekly growth trajectories for *C. sativa* and *B. vulgaris* extracted from automated ToF monitoring.

Weekly growth trajectories extracted from automated ToF monitoring are presented below in Figure 11, with RGB imagery confirming the measured trends. *C. sativa* exhibited accelerating vegetative growth, increasing from 21.6 mm (Week 1) to 565.6 mm (Week 5) at an average rate of 136.0 mm·week^−1^. *B. vulgaris* showed steady vertical development of 123.6 mm over the cultivation period at an average rate of 30.9 mm·week^−1^. During the *C. sativa* experiment, seedlings were transplanted from 40 × 40 × 40 mm Rockwool cubes [55], into 150 × 150 × 150 mm cubes [56]. Similarly, *B. vulgaris* pots were repositioned during week 3 of cultivation. Despite these interventions, the automated monitoring system maintained consistent tracking of growth progression.

### 3.5. Environmental Data Collection and Management

**Figure 12:**
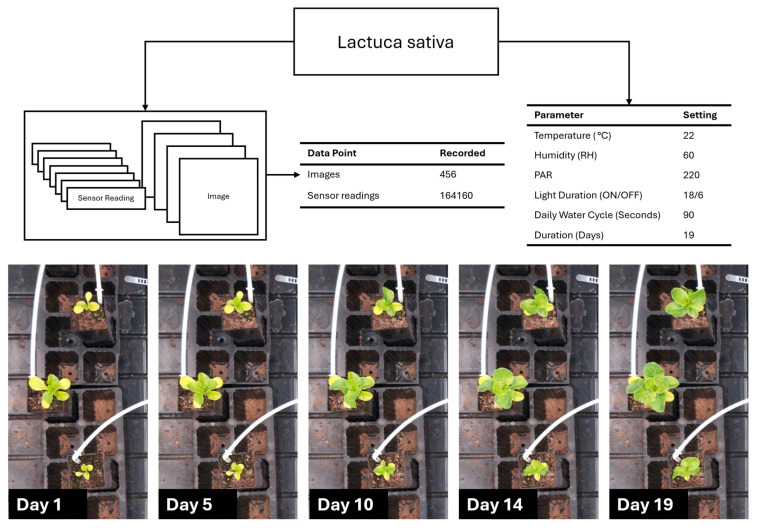
*Lactuca sativa* data collection, environment, and growth progress over 19 days.

Figure 12 presents the data collection performance during *L. sativa* cultivation (Section 2.5). The data collection system captured 456 images (one per hour) and 164,160 total environmental sensor readings (which were taken at 10 s intervals) throughout the 19-day experimental period. Total data storage was approximately 4.1 GB, comprising sensor readings in CSV format (~12.6 MB), RGB images in lossless PNG format (~4.07 GB) and ToF amplitude images also in PNG format (~9.1 MB). All data streams were successfully logged and transferred to the central server. The sequential imagery documented progressive vegetative development with observable increases in plant size, leaf expansion, and biomass accumulation.

### 3.6. Power Consumption

Table 3 presents the power consumption results as described in Section 2.8. System baseline power draw (Raspberry Pi controllers, sensors, communications) was 68 W. Lighting power consumption scaled from 334 W at minimum activation threshold (83 μmol·m^−2^·s^−1^) to 1936 W at full intensity (1241 μmol·m^−2^·s^−1^). Climate control power consumption varied by operating mode: cooling (658 W), heating (595 W), and fan only (32 W). Thermal efficiency was 0.033 °C·min^−1^·kW^−1^·m^−3^ for cooling and 0.043 °C·min^−1^·kW^−1^·m^−3^ for heating.

Maximum combined system draw during full-intensity lighting with active cooling was 2594 W. Fan-only mode consumed 4.9% of the power required for active cooling.

## 4. Discussion

The experimental results confirm the capacity of the MCSGC platform to deliver programmable and stable control across climate, lighting, and irrigation subsystems while supporting automated data collection and plant growth monitoring.

The climate control results demonstrate that the MCSGC can provide sufficient range and precision for a wide variety of phenotyping experiments. The operational range (11.7–38.6 °C) and the precision achieved covers the requirements for many crop species, enabling both moderate cool-season experiments and higher temperature stress studies. Precision slightly increased at higher temperatures suggesting that the system can reliably support heat-stress protocols that require precise elevated temperatures.

The ability of the platform to track a dynamic temperature recipe with a rapid response time highlights its value for more realistic climate research. By being able to simulate diurnal fluctuations and sudden weather events, the system enables more realistic environmental stress studies than static GCs. Energy efficient operation, achieved by the system predominately operating in fan mode (82.9% of the test), improves feasibility for many-cabinet large-scale experiments. Overall, these results confirm that the MCSGC can reproduce both stable and dynamic climates with research-grade accuracy, representing a significant advance over existing static setpoint systems.

The lighting test demonstrated that the platform can provide precise and predictable control over a wide variety of plant-growth requirements, from low-intensity shade-tolerant species to high-intensity conditions required for flowering crops. Unlike conventional GC’s limited to binary on/off fixed photoperiods, the programmable recipe system enables simulation of sunrise/sunset, cloud cover, and geographically specific lighting scenarios. This flexibility allows researchers to design experiments that more closely mirror natural light environments, supporting investigations into genotype-specific lighting responses. The high level of control reduces experimental uncertainty, as light treatments can be reliably reproduced across multiple trials and cabinets. Low-cost distributed multispectral sensing [57] could further extend these capabilities by enabling spatially resolved light monitoring across the growth area.

The irrigation subsystem tests demonstrated the capacity for consistent, precise delivery of water volumes, providing researchers with reliable control over one of the most critical variables in plant studies. This precision allows for fine-tuned drought stress protocols, water-use efficiency experiments, and reproducible experiments across multiple cabinets. By integrating the irrigation, temperature, and lighting into a unified “recipe” framework the ability to test complex interactions between these three critical factors for plant growth is simplified. However, the current system maintains fixed flow rates during individual experiments. While different precipitation intensities can be implemented by selecting alternative dripper specifications between experiments, real-time flow rate modulation is not currently supported. Despite this limitation, the system’s demonstrated consistency and integration with other environmental controls significantly broadens the scope of experiments that can be conducted in a GC, supporting more ecologically relevant and translatable phenotyping research.

The cultivation trials confirm that the MCSGC is capable of supporting species with markedly different physiological requirements while maintaining precise environmental control (±0.76 °C mean tracking error during dynamic temperature recipes; ±0.09 L·h^−1^ irrigation consistency) and simultaneously producing structured datasets suitable for AI analysis. The ToF imaging coupled with RGB imaging enabled automated extraction of morphological parameters including plant height, canopy volume, and leaf area, reducing manual measurement and associated subjectivity. This capability captured distinct growth dynamics, with rapid vegetative expansion in *C. sativa* (136.0 mm·week^−1^) compared to steady rosette development in *B. vulgaris* (30.9 mm·week^−1^), representing a 4.4-fold difference in growth rate quantified without manual intervention. While certain technical limitations emerged, such as camera position changes being potentially required for especially tall species, these represent engineering challenges that can be resolved through refinement rather than requiring full redesigns.

Additionally, the validation experiments relied on factory calibration and initial cross-calibration of environmental sensors without periodic recalibration during trials. While sufficient for demonstrating platform functionality over the 19–36-day experimental periods, long-term deployments would benefit from scheduled recalibration protocols, particularly for CO_2_ and PAR sensors.

The automated data pipeline test demonstrates how the MCSGC platform can reliably and robustly combine the phenotyping and environmental data into a single dataset, creating AI-ready datasets without the need for extensive post-processing for correlation. This lays the groundwork for future predictive modelling research and can even support the development of crop-science digital twin research.

Power consumption experiments demonstrated the platform’s potential for energy-efficient operation. Fan-only mode (32 W) consumed just 4.9% of the power required for active cooling (658 W), enabling substantial energy savings during stable temperature periods. During night periods (lights off), the system typically consumed 100 W (baseline plus fan-only mode), with a maximum of 726 W during active cooling. During daylight periods at 50% lighting intensity, typical consumption was 1353 W, increasing to 1979 W with active cooling. The absolute maximum system draw at full lighting intensity with active cooling was 2662 W. During dynamic climate simulation, the system operated in fan-only mode for 83.6% of the test period, with active cooling or heating required only during setpoint transitions. Lighting power scales linearly with intensity, enabling researchers to balance PAR requirements against energy costs. Combined with the modular design and low capital cost (<$10,000 AUD), these operational efficiencies further reduce barriers to entry for institutions seeking accessible phenotyping infrastructure.

When benchmarked against other phenotyping platforms, the MCSGC occupies a unique position. Commercial high-throughput imaging systems such as PlantScreen and LemnaTec Scanalyzer employ conveyor-based architectures optimised for imaging throughput but require separate growth infrastructure [58,59] and substantial investment (€60,000–3,000,000 [29]). Open-source solutions such as PhenoBox (€3000 [60]) and Phenotiki (€200 [61]) provide affordable imaging but are designed as add-ons to existing growth chambers, lacking environmental control integration. Conversely, commercial growth chambers like the Conviron PGW40 [24] provide environmental control but without integrated phenotyping or dynamic climate simulation. The MCSGC uniquely combines dynamic environmental control, automated phenotyping, and multi-cabinet coordination at low cost (<$10,000 AUD).

Compared to other GC platforms, the MCSGC achieved temperature control comparable to commercial systems despite operating under more demanding dynamic conditions. Commercial growth chambers specify static setpoint accuracy of ±0.3 °C (PHCbi MLR-352H [62]), ±0.5 °C (Percival PGC-105 [63]; Conviron PGW40 [24]), while Ariella et al. [20] reported ±1 °C in their research chamber within a narrow 24–27 °C range. The MCSGC maintained ±0.76 °C mean tracking error during dynamic climate simulation across a 15–31 °C range with continuously changing setpoints a substantially more demanding control scenario than static operation. Under comparable static conditions, the MCSGC demonstrated ±0.32 °C (at 23 °C) and ±0.07 °C (at 38 °C), matching or exceeding commercial specifications. The operational temperature range (11.7–38.6 °C) approaches commercial systems (10–45 °C in [24]) at a fraction of the cost. The ability of the platform to follow dynamic environmental recipes provides experimental flexibility not available in any system reported in the literature [19,20,21,22,23], while the server–client architecture offers multi-cabinet coordination for replicated, large-scale, and multi-environment studies critical for robust phenotyping. Together with the low-cost (<$10,000 AUD), modular design, these characteristics position the MCSGC as a significant advancement in both GC and automated phenotyping systems.

## 5. Conclusions

This paper presents a microclimate-controlled smart growth cabinet platform that bridges the gap between manual phenotyping and expensive commercial systems through four integrated innovations: sub-AUD $10,000 modular construction, multi-cabinet coordination, dynamic environmental recipes, and automated data synchronisation between environmental and phenotyping systems.

Testing confirmed that environmental control achieved 67.1% of measurements within ±1 °C during dynamic climate simulation (11.7–38.6 °C range), linear lighting response (R^2^ = 0.9999, 83–1241 μmol·m^−2^·s^−1^), precise irrigation (±0.09 L·h^−1^), and energy-efficient operation with thermal efficiency of 0.033–0.043 °C·min^−1^·kW^−1^·m^−3^ and typical power consumption of 1353 W at 50% lighting intensity (1979 W maximum with active cooling). Experiments also confirmed system performance through supporting the cultivation of three plant species with contrasting requirements in initial validation trials. Replicated biological studies represent the next research phase to quantify how controlled environmental variations affect phenotypic responses. Automated monitoring captured species-specific growth patterns (*C. sativa*: 544.1 mm over 34 days; *B. vulgaris*: 123.6 mm over 36 days; *L. sativa*: 19-day cultivation) while generating comprehensive datasets (456 images, 164,160 sensor readings) without manual intervention.

Future development should prioritise hardware extensions for temperatures below 10 °C operation, motorised camera positioning, substrate moisture sensing, and scheduled sensor recalibration protocols to expand experimental capabilities and ensure long-term measurement accuracy. Integration of soil moisture sensors would enable closed-loop irrigation control, automated detection of water excess, and more sophisticated drought stress protocols to enhance climate-resilience phenotyping research. Cloud-based architecture could also be integrated to improve long-term scalability.

By reducing barriers of access to advanced phenotyping infrastructure, this platform facilitates broader participation in developing climate-resilient crops. As environmental volatility increases, affordable systems that produce machine learning-compatible datasets become essential infrastructure for ensuring global food security through accelerated crop improvement.

## Figures and Tables

**Table 1:** Comparison of current growth cabinets.

Reference	System Scale	Environmental Control	Environmental Monitoring	Automation Level	Control Type	Scalability	Cost Category
Da Silva and Machado [19]	3.5 m × 1.1 m × 1.99 m; soybean, cotton, bean, tobacco	Humidity, water temperature, misting intervals	Temperature, humidity (precision not specified)	Manual data collection only	Static setpoints, manual adjustment	Greenhouse-dependent, non-modular	Low cost
Ariella et al. [20]	1 m × 0.6 m × 0.85 m; *Capsicum frutescens*	Temperature (25–27 °C), humidity (60–80% RH), soil moisture, lighting	±1 °C temperature, ±6% RH, imaging sensors	Automated sensor/image logging	Static setpoints, manual adjustment	Fixed single-unit design	Low cost
He et al. [21]	1.5 m × 1.5 m × 2.0 m; leafy vegetables	Temperature, humidity, CO_2_, light intensity, air circulation	Temperature, humidity, CO_2_, PPFD, wind speed (precision not specified)	Sensor monitoring, no vision system	Static setpoints, manual adjustment	Non-modular, single unit	Not specified
Hatzopoulos et al. [22]	Unspecified; *Capsicum annuum*	Temperature, humidity, light spectrum/intensity, CO_2_, soil moisture, VPD	Multi-parameter sensing, plant height via imaging	Automated data/image storage, height measurement	Static setpoints, manual adjustment	Limited removable sensors only	Low cost
Lee et al. [23]	Compact (unspecified); lettuce, pak choi	Temperature, light intensity, nutrient delivery	Temperature, humidity, CO_2_, canopy imaging	Vision-based phenotyping, automated logging	Static setpoints, manual adjustment	Single unit, non-expandable	Low cost
Conviron PGW40 [24]	3.86 m^2^ growth area, 1.93 m height; mature tall plants	Temperature (10–45 °C), multi-level lighting, airflow, fresh air	Temperature, pressure monitoring	Programmable alarms, monitoring systems	Manufacturer-programmed profiles	Commercial single unit	High cost (€60,000+)

**Table 2:** Controlled environment parameters for experimental cultivation.

Parameter	*Cannabis sativa*	*Beta vulgaris* subsp. *vulgaris*	*Lactuca sativa*
Temperature (°C)	24	18	22
Relative Humidity (%)	50	65	60
Light Cycle (h)	18/4 (light/dark)	16/8(light/dark)	16/8 (light/dark)
Light Intensity (µmol·m^−2^·s^−1^)	600	200	220
Water Cycle (day^−1^)	4	Manual	2
Water Duration (s)	60	Hand Watered	45
Growth Duration (d)	34	36	19
Growth Medium	Rockwool	Soil	Soil

**Table 3:** Power consumption across system operating conditions.

Subsystem	Condition	Current (A)	Voltage (V)	Power (W)
System baseline	Controllers, sensors, communications	0.30	226	68
Lighting	Minimum threshold (83 μmol·m^−2^·s^−1^)	1.48	226	334
Lighting	25% intensity	4.42	227	1003
Lighting	50% intensity	5.52	227	1253
Lighting	75% intensity	6.83	226	1543
Lighting	100% intensity (1241 μmol·m^−2^·s^−1^)	8.68	223	1936
Climate control	Cooling	2.90	227	658
Climate control	Heating	2.63	226	595
Climate control	Fan only	0.14	227	32

## Data Availability

The data presented in this study are available on request from the corresponding author.

## Supplementary Materials

The following supporting information can be downloaded at https://www.mdpi.com/article/10.3390/s25247509/s1, Figure S1: Growth Cabinet server architecture; Table S1: Sensors used in the Microclimate-Controlled Smart Growth Cabinet (MCSGC) system for environmental monitoring; Table S2: Vision-based sensors.

## Abbreviations

The following abbreviations are used in this manuscript:

AI	Artificial Intelligence
BOM	Bureau of Meteorology
CSI	Camera Serial Interface
CSV	Comma Separated Value
EC	Electrical Conductivity
GC	Growth Cabinet
HTTP	Hyper-Text Transfer Protocol
I^2^C	Inter-Integrated Circuit
LAI	Leaf Area Index
LED	Light Emitting Diode
MCSGC	Microclimate-Controlled Smart Growth Cabinet
MPC	Model Predictive Control
MQTT	Message Queuing Telemetry Transport
PAR	Photosynthetically Active Radiation
PCB	Printed Circuit Board
PID	Proportional-Integral-Derivative
POE	Power over Ethernet
PWM	Pulse Width Modulation
RH	Relative Humidity
RGB	Red, Green, Blue
SBC	Single-Board Computer
SPI	Serial Peripheral Interface
ToF	Time-of-Flight
USB	Universal Serial Bus
VPD	Vapour Pressure Deficit
*J*	Cost function
*R*^2^	Coefficient of determination
*T*	Temperature (°C)
*t*	Time (s)
*T*sp	Target setpoint temperature (°C)
*u*	Control signal
α	Learning rate
Δ*P*	Change in control action
Δ*t*	Time step (s)
