# Supplementary material for: Microclimate-Controlled Smart Growth Cabinets for High-Throughput Plant Phenotyping
Source: Sensors (Basel). 2025 Dec 10;25(24):7509. doi: 10.3390/s25247509 (PMC12737258; doi:10.3390/s25247509)
Supplement: Supplementary file 1 [file sensors-25-07509-s001.zip › sensors-3998118-supplementary.pdf]

sensors MDPI

1. Sensor and Camera Specifications

Table S1 summarises the sensors used in the MCSGC system, including their measured parameters, accuracy specifications, communication interfaces, and assigned subsystems.

Table S1 Sensors used in the Microclimate-Controlled Smart Growth Cabinet (MCSGC) system for environmental monitoring.

Sensor	Parameter	Accuracy/Features	Interface/Controller	Subsystem
SHT45 [34]	Temperature & Humidity	±0.1°C (temperature), ±1.0% RH (humidity)	I^2C	Internal (Cabinet)
LPS22HH [35]	Atmospheric Pressure	0.5 hPa	I^2C	Internal (Cabinet)
AS7341 [36]	Light Spectrum (multi-spectral intensity)	11 channels covering 350nm to 1000nm	I^2C	Internal (Cabinet)
EZO-CO_2 [37]	Carbon Dioxide (CO_2) Concentration	(+/- 5%) + (+/- 50 ppm)	I^2C via EZO board	Internal (Cabinet)
Atlas Mini Lab Grade pH Probe [38]	pH	+/– 0.002	Integrated via Atlas EZO i3 InterLink	External (Water Supply)
Atlas Mini Lab Grade EC Probe [39]	Electrical Conductivity (EC)	+/ – 2%	Integrated via Atlas EZO i3 InterLink	External (Water Supply)
Atlas Water Temperature Sensor PT-100 [40]	Water Temperature	+/- (0.15 + (0.002*t))	Integrated via Atlas EZO i3 InterLink	External (Water Supply)

Table S2 summarizes the vision-based sensors used for automated plant phenotyping, including resolution, interface, and measurement purpose.

Table S2 Vision-based sensors.

Device	Parameter/Measurement	Resolution/Specs	Interface/Controller	Purpose
Arducam RGB Camera [46]	High-resolution visual imagery	64 megapixels	Camera Serial Interface (CSI)	Capture detailed imagery of plant morphology and track growth patterns
Arducam ToF Camera [47]	Three-dimensional imaging (canopy height and spatial geometry)	240×180 pixels	CSI	Measure canopy height and assess 3D plant development dynamics
FLIR Lepton 3.5 Thermal Camera [48]	Thermal imagery (leaf temperature, stress detection)	160×120, 57° with shutter	Universal Serial Bus (USB)	Detect plant stress and monitor transpiration rates

2. Growth Server

The growth server acts as a central point of storage and control for the GCs. The architecture of the system enables precise control of experimental conditions while maintaining system scalability. This architecture facilitates management of multiple GCs from a central control system.

The server is setup to run locally on a computer running Ubuntu Server Ubuntu 24.04 with provisions to be deployable via cloud. This central server manages the entire experimental platform for multiple cabinets simultaneously, it serves as the primary interface between researchers and the physical GCs, providing comprehensive control and monitoring capabilities. The central controller is implemented using Python with a FastAPI

framework, interconnected with an SQLite database for storing environmental readings, experimental parameters, and image metadata, offering robust performance and flexible API integration.

2.1 Cabinet Communication and Protocol

The GC communicates with the central system through both HTTP and MQTT protocols. This bidirectional communication can be broken down into four primary functional areas: Unit Configuration & System Control, Recipe Data, Sensor and Image Data, and Heartbeat.

1. Unit Configuration stores cabinet-specific settings including sensor reading frequency, hardware identification, and control algorithm parameters.
2. System Control translates experimental parameters into hardware commands, implementing control loops for environmental systems and executing timing sequences for photoperiod and irrigation events.
3. Recipe Data provides dynamic setpoints programmable for each second of the day via CSV format. Two modes are supported: staged 24-hour cycles or date-time-based schedules for specific events. Recipes contain environmental conditions, lighting schedules, and irrigation timing parameters.
4. Sensor and Image Data is captured locally then synchronised with the server for backup. This dual-storage approach provides data protection whilst accommodating network interruptions and minimising bandwidth requirements.
5. The Heartbeat function maintains continuous communication between GC controllers and the central system, transmitting status updates and implementing recovery protocols during disruptions.

2.2 Server Data Handling and Labelling

Data quality is critical for plant research as it enables precise linkage between experimental conditions, environmental data, and plant traits. The system implements automated data handling with integrated metadata that ensures data integrity and accessibility throughout the research lifecycle as demonstrated in Figure S1.

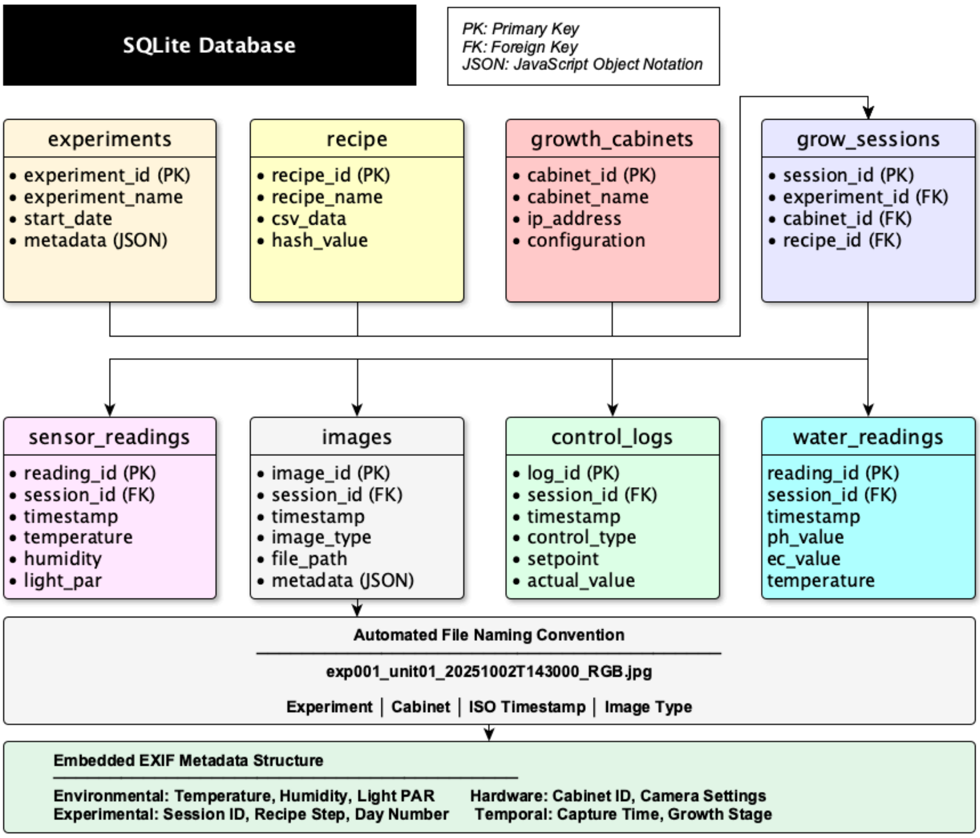

Figure S1 Growth Cabinet server architecture

The platform utilises structured filename conventions following the format ExperimentID_UnitID_ISOTimeStamp_ImageType. For example, exp001_unit01_20251002T143000_RGB.jpg identifies research project 001, growth unit 01, capture time (2 October 2025, 14:30:00), and image type (RGB). This human-readable format facilitates quick identification without requiring database queries.

Additionally, comprehensive metadata is automatically embedded within each data file (detailed structure provided in Supplementary Material), ensuring that environmental conditions remain permanently linked to phenotypic observations. This automated metadata integration eliminates manual data correlation processes while creating consistently formatted datasets suitable for machine learning applications.

This integrated data handling ensures that critical experimental context remains accessible even if files are relocated, creating AI-ready datasets that maintain their integrity throughout the research lifecycle and support future model development for agricultural applications
